# Is stereotactic radiosurgery a rational treatment option for brain metastases from small cell lung cancer? A retrospective analysis of 70 consecutive patients

**DOI:** 10.1186/s12885-015-1103-6

**Published:** 2015-03-04

**Authors:** Shoji Yomo, Motohiro Hayashi

**Affiliations:** 1 Division of Radiation Oncology, Aizawa Comprehensive Cancer Center, Aizawa Hospital, 2-5-1, Honjo, Matsumoto, Nagano 390-0814 Japan; 2 Saitama Gamma Knife Center, San-ai Hospital, Saitama, Japan

**Keywords:** Brain metastases, Small cell lung cancer, Stereotactic radiosurgery, Whole brain radiotherapy

## Abstract

**Background:**

Because of the high likelihood of multiple brain metastases (BM) from small cell lung cancer (SCLC), the role of focal treatment using stereotactic radiosurgery (SRS) has yet to be determined. We aimed to evaluate the efficacy and limitations of upfront and salvage SRS for patients with BM from SCLC.

**Methods:**

This was a retrospective and observational study analyzing 70 consecutive patients with BM from SCLC who received SRS. The median age was 68 years, and the median Karnofsky performance status (KPS) was 90. Forty-six (66%) and 24 (34%) patients underwent SRS as the upfront and salvage treatment after prophylactic or therapeutic whole brain radiotherapy (WBRT), respectively. Overall survival (OS), neurological death-free survival, remote and local tumor recurrence rates were analyzed.

**Results:**

None of our patients were lost to follow-up and the median follow-up was 7.8 months. One-and 2-year OS rates were 43% and 15%, respectively. The median OS time was 7.8 months. One-and 2-year neurological death-free survival rates were 94% and 84%, respectively. In total, 219/292 tumors (75%) in 60 patients (86 %) with sufficient radiological follow-up data were evaluated. Six-and 12-month rates of remote BM relapse were 25% and 47%, respectively. Six-and 12-month rates of local control failure were 4% and 23%, respectively. Repeat SRS, salvage WBRT and microsurgery were subsequently required in 30, 8 and one patient, respectively. Symptomatic radiation injury, treated conservatively, developed in 3 patients.

**Conclusions:**

The present study suggested SRS to be a potentially effective and minimally invasive treatment option for BM from SCLC either alone or after failed WBRT. Although repeat salvage treatment was needed in nearly half of patients to achieve control of distant BM, such continuation of radiotherapeutic management might contribute to reducing the rate of neurological death.

## Background

Lung cancer is the most common source of brain metastasis (BM). Given that the cumulative incidence of BM from small cell lung cancer (SCLC) at 2 years is approximately 50% [1], prophylactic cranial irradiation (PCI) combined with systemic chemotherapy, which moderately prolongs overall survival (OS) by reducing the incidence of delayed BM, has long been accepted as the standard of care for most patients [2-5]. Recurrence or progression of intracranial disease after such an intensive treatment regimen is, however, not uncommon despite the radiosensitive nature of SCLC [6]. The prognosis of patients with recurrent BM generally remains dismal.

Stereotactic radiosurgery (SRS) has emerged as the preferred treatment modality, either alone or in combination with other modalities. Recently, in selected patients, whole brain radiotherapy (WBRT) has been omitted from the initial management for BM with the aim of reducing the potential risk of delayed neurological toxicity [7,8]. Given the propensity for dissemination of SCLC, SRS does not appear to be a rational approach to this malignancy. To date, there have been only a few, relatively small, studies of SRS for SCLC with or without prior WBRT (Table 1) [9-13]. Thus, the role of focal treatment by means of SRS for BM from SCLC remains to be elucidated.

**Table 1:** **Outcomes of patients undergoing SRS for BM from SCLC**

First author & year	Treatment modality	No. of Patients	No. receiving prior WBRT (%)	MST after SRS (months)	Local tumor control	Remote brain recurrence
Wegner 2011 [9]	GK	44	30 (68)	9	90%/1 year	61%/7 months
Jo 2011 [12]	GK	50	38 (76)	*6.3	70.3%/5.6 months	29.7% (crude)
Harris 2012 [13]	GK	51	34 (67)	5.9	57%/1 year	58%/1 year
Olson 2012 [10]	CK	27	19 (70)	3	76.5/1 year	60%/3.5 months
Nakazaki 2013 [11]	GK	44	44 (100)	5.8	95.8%/4 months	50%/6 months
Present study 2014	GK	70	24 (34)	7.8	77%/1 year	47%/1 year

SRS stereotactic radiosurgery, BM brain metastasis, SCLC small cell lung cancer, WBRT whole brain radiotherapy, MST median survival time, GK gamma knife, CK cyberknife, *mean value.

We retrospectively investigated the efficacy and limitations of our SRS-oriented treatment strategy for patients with newly diagnosed and recurrent BM from SCLC.

## Methods

### Patient population

The present study was conducted in compliance with the Declaration of Helsinki (6th revision, 2008), and fulfilled all of the requirements for patient anonymity. The San-ai Hospital Institutional Review Board approved this retrospective clinical study in January 2014. Between January 2009 and October 2013, 70 consecutive patients with BM originating from histologically proven primary SCLC underwent Gamma Knife SRS in our institution. Fifty-five patients were male and 15 were female. The median age was 68 years (range: 44–85 years). The median Karnofsky performance status (KPS) at the time of SRS was 90 (range: 30–100). Before SRS, 7 patients had undergone microsurgical resection for BM and one had received third ventriculostomy for obstructive hydrocephalus. Prior WBRT had been conducted at the referring regional hospitals, prophylactically in 7 patients and in a therapeutic setting in 16. One patient had undergone hypofractionated radiotherapy for a large tumor located in the posterior cranial fossa. All patients with prior WBRT had documented intracranial failure (either new lesions or progression of preexisting metastases). The median interval between primary diagnosis and SRS was 11.4 months (range: 0.1–150 months). Patient characteristics are summarized in Table 2.

**Table 2:** **Summary of clinical data from 70 consecutive patients**

Characteristics	Overall (n=70)
Sex (male/female)	55/15
Age (years), median (range)	68 (44–85)
KPS, median (range)	90 (30–100)
Active extra-CNS disease	45 (64%)
Prior WBRT	24 (34%)
Post-SRS chemotherapy	50 (71%)
Time from primary diagnosis to initial SRS (months), median (range)	11.4 (0.1–150)
Cumulative PTV on initial SRS (mL), median (range)	4.4 (0.5–50.3)
No. of intracranial lesions on initial SRS, median (range)	2 (1–21)

KPS Karnofsky performance status, CNS central nervous system, WBRT whole brain radiotherapy, SRS stereotactic radiosurgery, PTV planning target volume.

### Radiosurgical indications and techniques

All patients included in the present study had been diagnosed and their primary tumors treated at the referring regional hospitals, whose own cancer boards had provisionally determined the appropriateness of SRS. The patients were then referred to our institution to receive SRS for BM. The SRS protocol used in this study was based on the standard care established at our institution. In the upfront setting, patients with up to ten BM principally received SRS. When abnormal enhancement of cranial nerves, the ventricular ependymal layer and/or the cortical surface or more than 10 BM were documented by high resolution magnetic resonance (MR) imaging at the time of initial SRS, WBRT was recommended. In the salvage setting, the treatment protocol in the author’s institution has no set limit on the number of BM. Providing that WBRT had either already been performed or refused by the patient, SRS was applied for multiple BM, even in cases with more than 10 lesions, when the patient’s systemic condition was such that SRS intervention would be tolerable and fully informed consent for treatment had been obtained. Surgical resection was, in principle, indicated for large tumors (≥10 mL) with a mass effect unresponsive to corticosteroid therapy. If surgery did not seem feasible due to a poor prognosis or advanced systemic disease, 2-session SRS was indicated for carefully selected large tumors (≥10 mL) [14].

SRS was performed using the Leksell G stereotactic frame (Elekta Instruments, Stockholm, Sweden). The frame was placed on the patient’s head under local anesthesia supplemented with mild sedation. Three-dimensional volumetric gadolinium-enhanced T1-weighted MR images, 2 mm in thickness T2-weighted MR images and contrast-enhanced computed tomography covering the whole brain were routinely used for dose planning with Leksell Gamma Plan software (Elekta Instruments). When performing salvage SRS after prior WBRT, the targets were limited to recurrent or newly emerging lesions. Stable lesions continued to be monitored unless regrowth was documented. Prescribed doses were selected in principle according to the dose protocol of the JLGK 0901 study [15], though a margin of approximately 1 to 2 mm was added to the visible lesion in consideration of the infiltrative nature of SCLC [16]. The technical details of 2-session SRS were previously described in detail [14]. All treatments were performed with the Leksell Gamma Knife Model C or Perfexion.

### Post-SRS management and follow-up evaluation

Clinical follow-up data as well as contrast-enhanced MR images were obtained every one to three months. If metachronous remote metastases were identified, they were, in principle, managed with repeat SRS. When miliary metastases (numerous tiny enhanced lesions) and/or leptomeningeal carcinomatosis was newly documented, WBRT was then considered unless it had been used previously. Local control failure was defined as an at least 20% increase in the diameter of the targeted lesions, taking as a reference the pre-SRS diameter, irrespective of whether the lesion was a true recurrence or delayed radiation injury. Delayed radiation injury was differentiated from tumor recurrence using serial MR imaging [17] and, in selected cases, ^11^C-methionine positron emission tomography. Additional SRS was possible provided that the volume of the local tumor recurrence was small enough for single-dose SRS. Surgical removal was indicated when neurological signs became refractory to conservative management, regardless of whether the radiological diagnosis was local tumor progression or radiation necrosis. Any adverse events attributable to SRS procedures were evaluated based on the National Cancer Institute Common Terminology Criteria for Adverse Events (NCI-CTCAE; ver.3.0). Before closing the research database for analysis, the authors updated the follow-up data of patients who had not visited our outpatient department for more than two months. Inquiries about the date and mode of death were made by directly corresponding with the referring physician and/or the family of the deceased patient, with written permission obtained at the time of undertaking SRS from all patients and/or their relatives, allowing the use of personal data for clinical research. Neurological death was defined as death attributable to central nervous system (CNS) metastases including tumor recurrence and carcinomatous meningitis.

### Statistical analysis

The overall survival (OS) rate was calculated by the Kaplan-Meier product limit method. The neurological and non-neurological death rates were calculated employing Gray’s test [18], wherein each event was regarded as a competing risk for another event. For the estimation of local control failure rates and distant BM recurrence, Gray’s test was similarly used, with subsequent WBRT for remote recurrence and the patient’s death being regarded as competing events, respectively. All of the above analyses were based on the interval from the date of initial SRS treatment until the date of each event. The Cox and Fine-Gray proportional hazards models [19] were employed to investigate prognostic factors for OS and neurological death-free survival, and for local tumor control, respectively. Potential prognostic factors were selected with reference to other SRS series [9-13]. The survival results were tested employing two prognostic scoring systems validated for SCLC (Diagnosis-specific graded prognosis assessment (DS-GPA) and Rades’s survival score). The statistical processing software package “R” version 3.0.1 (The R Foundation for Statistical Computing, Vienna, Austria) was used for all statistical analyses. A *P*-value of < 0.05 was considered to indicate a statistically significant difference.

## Results

SRS was conducted as an initial treatment in 46 patients (66%) and as salvage in 24 (34%). Forty-five patients (64%) had active systemic disease and/or extra-CNS metastases and 50 patients (71%) were still receiving systemic chemotherapy at the time of the initial SRS. In total, 292 tumors were being treated at the time of the initial SRS. The median planning target volume (PTV) was 0.60 mL (range: 0.04–22.3 mL). The median number of BM at SRS was 2 (range: 1–21 tumors) and the median cumulative PTV was 4.4 mL (range: 0.5–50 mL). Prescribed doses ranged from 12 Gy to 22 Gy (median: 20 Gy). Seven patients with large tumors were allocated to 2-session SRS.

**Figure 1:**
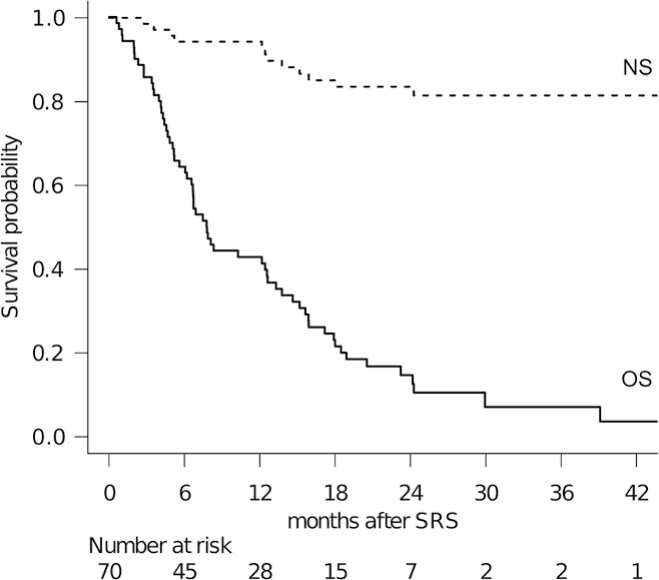
**Survival results for patients with BM from SCLC treated with SRS.** The solid line represents overall survival (OS) probability. The median survival time (MST) was 7.8 months (95% CI: 6.2–12.6). One-and 2-year OS rates after SRS were 43% and 15%, respectively. The dotted line represents the neurological death-free survival (NS) probability adjusted for competing events. The 1-and 2-year NS rates after SRS were 94 and 84%, respectively. Note that the distance between these two lines, NS and OS, represents the cumulative incidence of non-neurological death.

Full clinical results were available for all 70 patients as none were lost to follow-up. The median follow-up time after SRS was 7.8 months (range: 0.6–56 months). At the time of assessment, 8 patients (11%) were alive and 62 (89%) had died. The causes of death were intracranial local progression in 3 cases, meningeal carcinomatosis in 9 and progression of the primary lesion in 50. The 1-and 2-year OS rates after SRS were 43% and 15%, respectively (Figure 1). The median OS time was 7.8 months (95% CI: 6.2–12.6). The proportional hazards model for OS identified high KPS (HR: 0.493, 95% confidence interval (CI): 0.279–0.871, *P*=0.015) and solitary metastasis (HR: 0.419, 95% confidence interval (CI): 0.205–0.857, *P*=0.017) as favorable prognostic factors independently predicting OS rates (Table 3). One-and 2-year neurological death-free survival probabilities adjusted for competing events (non-neurological death) were 94% and 84%, respectively (Figure 1). The proportional hazards model suggested high KPS and no prior WBRT to be associated with lower risk of neurological death (Table 3), though neither reached statistical significance. The survival results were tested with validated prognostic scoring systems (Table 4). The DS-GPA showed significant differences in median survival time (MST): DS-GPA 3–4 points: 12.4 months (95% CI: 4.2-not reached), 1.5–2.5 points: 7.8 months (95% CI: 4.8–18.0), ≤1.0 points: 6.7 months (95% CI: 4.7–12.6) (*P*=0.036, log-rank test) (Table 4). A survival scoring system specifically for patients with BM from SCLC, as proposed by Rades et al. [20], also allowed stratification by 6-month patient survival rates: 15 points: 78% (95% CI: 51–91), 9–12 points: 66% (95% CI: 49–79), 5–8 points: 43% (95% CI: 18–66) (*P*=0.006, log-rank test) (Table 4).

**Table 3:** **Analysis of factors predicting patient survival after SRS (Proportional hazards model)**

Covariate	OS		NS	
	P value	Hazard ratio (95% CI)	P value	Hazard ratio (95% CI)
Young (≤65 years)	0.838	1.06 (0.612–1.83)	0.290	0.490 (0.131–1.83)
High KPS (≥90)	0.015	0.493 (0.279–0.871)	0.055	0.236 (0.054–1.03)
Controlled extra-CNS disease	0.365	0.709 (0.337–1.49)	0.150	2.44 (0.715–8.33)
Prior WBRT	0.629	0.868 (0.487–1.54)	0.071	3.81 (0.891–16.3)
Post-SRS chemotherapy	0.077	0.564 (0.299–1.06)	0.350	2.39 (0.383–14.9)
Single BM	0.017	0.419 (0.205–0.857)	0.340	1.69 (0.572–5.00)

SRS stereotactic radiosurgery, OS overall survival, NS neurological death-free survival, CI confidence interval, KPS Karnofsky performance status, WBRT whole brain radiotherapy, BM brain metastases.

**Table 4:** **Survival of patients with BM from SCLC stratified with prognostic classification systems**

	Survival results (No. of patients)	*P*value
Overall MST in months	7.8 (70)	
DS-GPA (MST in months)		0.036
0–1.0	6.7 (35)	
1.5–2.5	7.8 (27)	
3.0–4.0	12.4 (8)	
Rades’s survival score (6-month survival rate)		0.006
5–8	43% (14)	
9–12	66% (38)	
15	78% (18)	

BM brain metastases, SCLC small cell lung cancer, MST median survival time, DS-GPA diagnosis specific-graded prognosis assessment.

**Figure 2:**
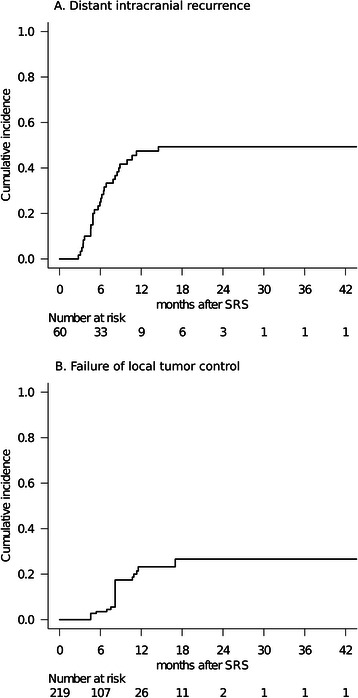
**Cumulative incidences of distant intracranial recurrence (A) and local tumor control failure (B).** The 6-and 12-month distant intracranial recurrence rates were 25% and 47%, respectively. The 6-and 12-month local tumor control failure rates were 4% and 23%, respectively.

Only the 219/292 tumors (75%) in 60 patients (86%) who had sufficient radiological follow-up data were analyzed herein because the other 10 patients died from extra-CNS progression without follow-up MR imaging. Remote metachronous BM were observed in 33 patients (55%). The 6-month and 1-year remote BM recurrence rates (per patient) after SRS were 25% and 47%, respectively (Figure 2A). The 6-month and 1-year local tumor control failure rates (per lesion) were 4% and 23%, respectively (Figure 2B). Twenty-three metastases were eventually diagnosed as local recurrence or delayed radiation injury at a median time of 8.2 months after SRS (range: 4.6–17 months). The proportional hazards model demonstrated low marginal dose (HR: 4.24 95% CI: 1.21–14.8, *P*=0.024) and prior WBRT (HR: 7.11 95% CI: 2.80–18.0, *P <* 0.001) to be factors predicting a higher local tumor control failure rate (Table 5). Two-session SRS conducted for large tumors achieved a durable tumor volume reduction coupled with symptom relief in 6 of 7 cases. One male patient with a large brainstem metastasis experienced local control failure, which eventually resulted in neurological death 12 months after SRS.

**Table 5:** **Analysis of factors predicting local tumor control failure (Proportional hazards model)**

Covariate	P value	Hazard ratio (95% CI)
Prior WBRT	< .001	7.11 (2.80–18.0)
Large target volume (>2 mL)	0.085	0.865 (0.193–3.88)
Tumor causing focal deficit	0.97	1.05 (0.104–5.14)
Low marginal dose (<20Gy)	0.024	4.24 (1.21–14.8)

CI confidence interval, WBRT whole brain radiotherapy.

Thirty patients (43%) required repeat SRS for remote or local BM recurrence. The total number of SRS sessions ranged up to 5 (median: 1) and the total number of BM treated per patient ranged up to 72 (median: 5). Eight patients (17%) without prior WBRT underwent salvage WBRT at a median time of 9.8 months after SRS (range: 2.8–22.6 months) because of subsequent development of miliary BM and/or leptomeningeal dissemination. Microsurgical resection was necessary for local tumor recurrence in one patient at 15 months after SRS.

None of the adverse effects observed in this series exceeded NCI-CTCAE grade 3 toxicity. Three patients required oral steroids coupled with hyperbaric oxygen therapy for delayed radiation injury (NCI-CTCAE Grade 3 toxicity) and eventually showed clinical and radiological stabilization.

## Discussion

Advances in the development of systemic treatments, together with judicious use of surgical resection, WBRT and SRS, have led to increases in the number of long-term survivors and the MST. The long-term control of CNS disease has become increasingly important not only for overall disease control but also for the patient’s quality of life. The risk of developing BM in SCLC is higher than with other histologies. Seute et al. reported the cumulative risk of BM at 2 years after the diagnosis to be 49% to 65% in SCLC [1]. Thus, PCI has long been advocated to reduce the incidence of BM development [2-5]. The survival advantage in previous randomized trials supporting PCI as the standard of care is widely recognized as level 1 evidence. This approach may, however, at least theoretically increase the potential risk of leukoencephalopathy in patients without any known intracranial disease, but with a 50% probability that at some point CNS disease will appear [21,22]. In addition, intracranial disease control failure will continue to occur despite the relatively radiosensitive nature of SCLC [3,4,23]. Certainly, WBRT only treats existing disease and there is no evidence indicating that PCI prevents new BM from developing in patients with active systemic disease.

SRS for BM from SCLC has been relegated to use mainly after failed WBRT probably due to lack of evidence of the efficacy of SRS for this malignancy [9-13]. However, recent refinements in diagnostic and therapeutic modalities may impact the modern management of BM. High-resolution neuroimaging such as 3-dimensional volumetric imaging and the 3-tesla unit might become routinely available for visualizing lesions that used to be undetectable with older imaging modalities [24,25]. Recent technological breakthroughs in the SRS apparatus [26] have made it possible to safely treat 20, or even more, BM, provided that the lesions are small, in a one-day session. The delivery of highly focused radiation with a sharp dose fall-off is theoretically expected to reduce delayed neurotoxicity, and this feature makes it applicable both in the upfront and the salvage setting after recurrence or progression after prophylactic or therapeutic WBRT. A recent Japanese multi-institutional prospective study including 1194 patients (76% with lung cancer) suggested that the upfront SRS strategy is reasonable for patients with up to 10 lesions [15]. However, a critical argument can be made that the pathology of SCLC is unsuitable for SRS because of the disseminated nature of this malignancy. Thus, in our view, the efficacy and limitations of a focal therapeutic approach for BM from SCLC have yet to be determined.

The survival results after SRS in the present study are comparable to those of previous studies [9-11,13] (Table 1). What makes the present study different from the previous series is the ratio of upfront to salvage intervention. Upfront treatment accounts for almost two-thirds of our cohort, while salvage treatments were most numerous in previous series. We had anticipated before this investigation that the survival results would be worse in patients undergoing salvage treatment than in those receiving upfront treatment, but there was, in fact, no significant difference between these two groups (Table 3). We speculate that this might, at least in part, be attributable to patients receiving SRS as salvage having been self-selected to do well by virtue of having had time to develop recurrent BM and not dying of their systemic disease. Patient survival could be stratified employing validated prognostic grading systems. The DS-GPA index is one of the most relevant diagnostic tools for predicting the survival of patients with newly diagnosed BM [27]. In the original DS-GPA study, where the majority of patients (82.6%) received WBRT as the sole treatment, the survival of those with newly diagnosed BM from SCLC was 4.9 months, which was worse than those for patients with tumors at other primary sites. If confined to DS-GPA scores not exceeding 1.0, the MST was as short as 2.8 months. Considering that half the patients had DS-GPA scores of 1 or less in our cohort, the survival outcomes after SRS appear to be acceptable. Rades’s survival scoring system [20] also predicted the survival rates in our cohort, with the survival rates in the present study being higher in the lower score classes than in the original dataset. With regard to prognostic factors, high KPS and solitary BM were associated with improved patient survival in multivariate analyses (Table 3). Both variables were actually incorporated into the above survival scoring systems and these findings were also reproduced in prior studies focusing mainly on salvage treatment [9,13]. Identifying prognostic factors for longer survival in patients with BM would be critically important for assigning patients to the optimal treatment modality. This observation suggests that selected subsets of patients can be expected to experience prolonged survival, though the expected survival of patients with BM from SCLC may be limited in the majority of cases.

In the curve of local tumor control failure, an irregular elevation was observed around 8 months after SRS (Figure 2B). We speculate that the following factors may account for this observation. In a male patient who had received WBRT for multiple BM, multiple recurrent tumors initially responded well to SRS but the enhancement subsequently enlarged in most of these lesions. They were eventually diminished again by salvage re-SRS. Considering that salvage was successful, these lesions should be regarded as true local recurrence. The reason for the higher rate of local tumor control failure in patients with prior WBRT demonstrated herein remains unknown. However, it might be attributable to selective regrowth of radio-resistant tumor cells or to the surrounding brain tissue being predisposed to radiation injury. Thus, we recommend a high marginal dose (≥20 Gy), when possible, being given even for recurrent BM after WBRT, by referring to the results of multivariate analysis for local tumor control (Table 5).

Nearly half of our patients eventually experienced metachronous recurrence outside the treated area after the initial SRS. Subsequent SRS was needed in as many as 30 patients (43%), mostly because of remote BM recurrence. These patients were successfully managed with minimal toxicity. Only eight patients without prior WBRT eventually underwent salvage WBRT because of miliary metastases or leptomeningeal dissemination. Considering that remote recurrence frequently developed, meticulous clinical and neuroimaging follow-up and salvage SRS in a timely manner should be considered essential for assuring the relevance of SRS management. Such a continued radiotherapeutic management protocol might contribute to reducing the neurological death rate, though OS results after SRS were comparable to those of previous studies (Figure 1). This finding is not consistent with the previous study by Harris et al. showing the rate of neurological death to be as high as 53% [13]. In our country, nation-wide availability of advanced diagnostic imaging facilities and radiosurgical equipment as well as the public healthcare system may, fortunately, be making it possible to provide cancer patients with easy access to necessary advanced medical services [28].

The present results must be interpreted with caution. Although the treatment results in our cohort suggested survival similar to that obtained with WBRT in properly stratified populations, a patient selection bias inherent to the retrospective approach is unavoidable. One of the critical issues in the present study is that the reason for PCI having been omitted could not be specified for all cases. It must be appreciated that we cannot address the potential role of SRS in comparison to WBRT because this was a small retrospective observational study. The survival advantage in previous randomized trials supporting PCI as the standard of care also cannot be ignored. The evidence for the clinical efficacy of SRS for BM from SCLC remains insufficient and more evidence-based information and additional research are needed to confirm the therapeutic benefits of SRS. We consider the present retrospective study to have been necessary as a means of hypothesis generation for future investigations.

## Conclusions

To our knowledge, this is the largest retrospective study investigating the efficacy of SRS for BM in patients with SCLC. Our results suggest SRS to be a potentially effective and minimally invasive treatment option for BM from SCLC either alone or after failed WBRT. Continued radiotherapeutic management might contribute to reducing the neurological death rate, though OS results after SRS were comparable to those of previous studies. SRS provided durable local tumor control, but repeat salvage treatment was needed in nearly half of patients to achieve control of distant BM.

## Abbreviations

BM: Brain metastases
SCLC: Small cell lung cancer
SRS: Stereotactic radiosurgery
KPS: Karnofsky performance status
WBRT: Whole brain radiotherapy
PCI: Prophylactic cranial irradiation
OS: Overall survival
MR: Magnetic resonance
NCI-CTCAE: National cancer institute common terminology criteria for adverse events
CNS: Central nervous system
DS-GPA: Diagnosis-specific graded prognostic assessment
PTV: Planning target volume
HR: Hazard ratio
CI: Confidence interval
MST: Median survival time
